# Screening of cell‐virus, cell‐cell, gene‐gene crosstalk among animal kingdom at single cell resolution

**DOI:** 10.1002/ctm2.886

**Published:** 2022-08-02

**Authors:** Dongsheng Chen, Zhihua Ou, Jiacheng Zhu, Haoyu Wang, Peiwen Ding, Lihua Luo, Xiangning Ding, Chengcheng Sun, Tianming Lan, Sunil Kumar Sahu, Weiying Wu, Yuting Yuan, Wendi Wu, Jiaying Qiu, Yixin Zhu, Qizhen Yue, Yi Jia, Yanan Wei, Qiuyu Qin, Runchu Li, Wandong Zhao, Zhiyuan Lv, Mingyi Pu, Boqiong Lv, Shangchen Yang, Ashley Chang, Xiaofeng Wei, Fengzhen Chen, Tao Yang, Zhenyong Wei, Fan Yang, Peijing Zhang, Guoji Guo, Yuejiao Li, Yan Hua, Huan Liu

**Affiliations:** ^1^ BGI‐Shenzhen Shenzhen China; ^2^ Suzhou Institute of Systems Medicine Suzhou Jiangsu China; ^3^ Shenzhen Key Laboratory of Unknown Pathogen Identification BGI‐Shenzhen Shenzhen China; ^4^ College of Life Sciences University of Chinese Academy of Sciences Beijing China; ^5^ The MOE Frontier Science Center for Brain Research and Brain‐Machine Integration School of Brain Science and Brain Medicine Zhejiang University Hangzhou China; ^6^ Department of Physiology, School of Basic Medical Sciences Binzhou Medical University Yantai China; ^7^ School of Basic Medicine Qingdao University Qingdao China; ^8^ College of Life Sciences Zhejiang University Hangzhou China; ^9^ China National GeneBank Shenzhen China; ^10^ Center for Stem Cell and Regenerative Medicine Zhejiang University School of Medicine Hangzhou China; ^11^ Guangdong Provincial Key Laboratory of Silviculture Protection and Utilization Guangdong Academy of Forestry Guangzhou China

**Keywords:** crosstalk, single cell sequencing

## Abstract

**Background:**

The exact animal origin of severe acute respiratory syndrome coronavirus 2 (SARS‐CoV‐2) remains obscure and understanding its host range is vital for preventing interspecies transmission.

**Methods:**

Herein, we applied single‐cell sequencing to multiple tissues of 20 species (30 data sets) and integrated them with public resources (45 data sets covering 26 species) to expand the virus receptor distribution investigation. While the binding affinity between virus and receptor is essential for viral infectivity, understanding the receptor distribution could predict the permissive organs and tissues when infection occurs.

**Results:**

Based on the transcriptomic data, the expression profiles of receptor or associated entry factors for viruses capable of causing respiratory, blood, and brain diseases were described in detail. Conserved cellular connectomes and regulomes were also identified, revealing fundamental cell‐cell and gene‐gene cross‐talks from reptiles to humans.

**Conclusions:**

Overall, our study provides a resource of the single‐cell atlas of the animal kingdom which could help to identify the potential host range and tissue tropism of viruses and reveal the host‐virus co‐evolution.

## Graphical abstract

Single‐cell atlases of organs and PBMCs in 20 species were constructed. The expression patterns of viral receptors from 24 virus families were characterised in different tissues or PBMC samples to generate a conclusive view on the cellular tropism of viruses. An exploration of cellular communication in brain cells within 11 species was conducted.

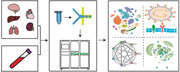

## 1 INTRODUCTION

The coronavirus disease 2019 (COVID‐19) caused by SARS‐CoV‐2 has rapidly surged around the world.^1^, ^2^ Understanding the viral host range is essential to prevent cross‐species transmissions of zoonotic viruses like SARS‐CoV‐2 and lots of efforts have been made to predict the potential zoonosis hotspots.^3^, ^4^, ^5^ Investigations on the host range of viruses using experimental methods can be restricted due to the unavailability of virus/animal/experimental resources, especially for pathogens with high biosafety risks. On the other hand, modelling analysis based on the phylogenetic, ecological and life‐history traits of hosts and viruses are able to pinpoint animal species or pathogens that are more prone to cause zoonosis. The combination of experimental evidence and modelling may greatly enhance our preparedness for zoonosis.^3^, ^6^, ^7^ It is known that host cellular surface proteins/ligands are employed by distinct pathogens as their receptors to initiate attachment and penetrate the cell. For example, angiotensin‐converting enzyme 2 (ACE2) for severe acute respiratory syndrome coronavirus (SARS‐CoV) and SARS‐CoV‐2,^8^, ^9^ and DPP4 for Middle East respiratory syndrome coronavirus (MERS‐CoV).^10^ Thus, the viral receptor distribution may reveal the potential replicating niches of the viruses. Transcriptome studies can identify the cellular receptor profiles for the virus of interest, which may be an effective way to narrow down the suspected host and tissue list.^11^ Identifying the host and tissue tropism is the first step towards understanding viral infection and pathogenesis, thus laying the foundation for the prevention and control of putative outbreaks in animals or humans. scRNAseq data can be utilised to explore conserved or divergent cell‐cell and gene‐gene cross‐talks among multiple species,^12^, ^13^, ^14^, ^15^ resulting in various molecular circuits important in development and diseases. In this study, we constructed the single cell atlas for 20 species and characterised the expressions of virus receptors at an unprecedented scale over 75 data sets among 44 animal species. Additionally, we dissected the connectome and regulome of neural cells based on brain single cell transcriptome data of multiple species, thus portraited a comprehensive communication atlas at cellular and genetic level.

## 2 RESULTS

### 2.1 Generation of single cell atlas for organs and peripheral blood mononuclear cells (PBMCs)

**FIGURE 1:**
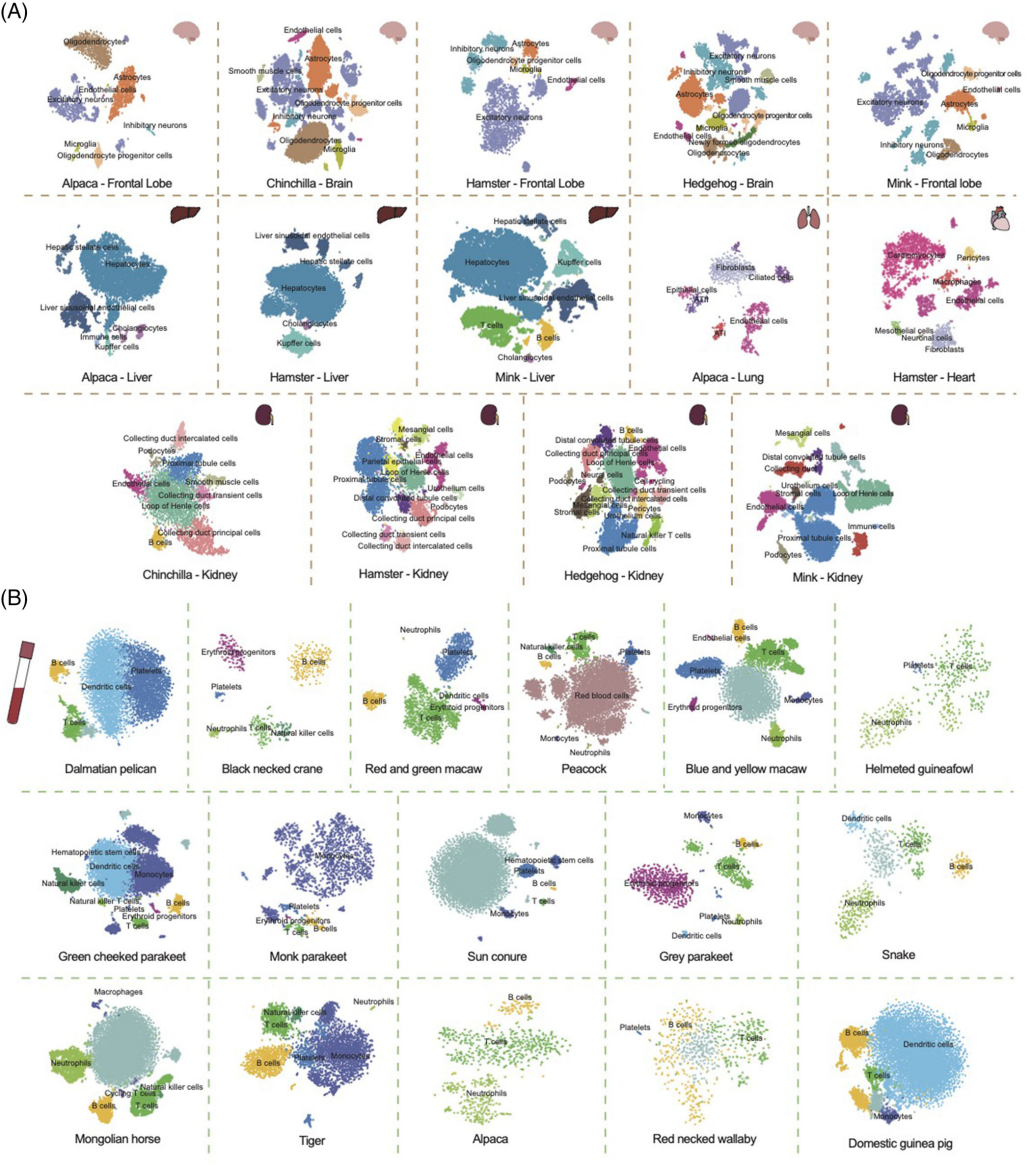
Generation of single‐cell atlases of 20 species. (A) tSNE plot of hedgehog (brain and kidney), alpaca (frontal lobe, liver and lung), mink (frontal lobe, liver and kidney), hamster (frontal lobe, liver, kidney and heart) and chinchilla (brain and kidney). (B) The PBMC atlases of dalmatian pelican, black necked crane, red and green macaw, peacock, blue and yellow macaw, helmeted guineafowl, green cheeked parakeet, monk parakeet, sun conure, grey parakeet, snake, horse, tiger, alpaca, red necked wallaby and domestic guinea pig.

Herein, we constructed the single cell atlas for a total of 20 species (Figure 1, Table S1). The multi‐organ atlases were comprised of hedgehog (brain and kidney), alpaca (frontal lobe, liver and lung), mink (frontal lobe, liver and kidney), hamster (frontal lobe, liver, kidney and heart) and chinchilla (brain and kidney) (Figure 1A). The PBMC atlases covered 5 mammal species (horse, tiger, alpaca, red necked wallaby and domestic guinea pig), 1 reptile species (snake) and 10 bird species (dalmatian pelican, black necked crane, red and green macaw, peacock, blue and yellow macaw, helmeted guineafowl, green cheeked parakeet, monk parakeet, sun conure and grey parakeet) (Figure 1B). After quality control (QC), 213 500 and 79 761 cells were maintained for downstream analysis. Cell identity for each cluster was assigned based on the specific expression of canonical cell type markers and the enrichment of GO terms (Tables S1–S, 3). The brain atlas mainly contained excitatory neurons, inhibitory neurons, microglia, oligodendrocytes, oligodendrocyte progenitor cells, astrocytes and endothelial cells. The kidney atlas was mainly composed of proximal tubule cells, loop of Henle cells, collecting duct principal cells, collecting duct intercalated cells, collecting duct transient cells, podocytes, pericytes, distal convoluted tubule cells, endothelial cells, smooth muscle cells and B cells. The lung atlas included type Ⅰ alveolar cells (ATI), type II alveolar cells (ATII), ciliated cells, endothelial cells, epithelial cells and fibroblasts. The heart atlas contained neuronal cells, cardiomyocytes, fibroblasts, mesothelial cells, macrophages, endothelial cells and pericytes. The liver atlas contained liver sinusoidal endothelial cells, cholangiocytes, Kupffer cells, hepatocytes and hepatic stellate cells. The major immune cells, including T cells, B cells, macrophages, natural killer cells and dendritic cells, were identified in PBMC atlas (Figure 1B).

### 2.2 Screening of entry factors for coronaviruses in 27 mammalian species

**FIGURE 2:**
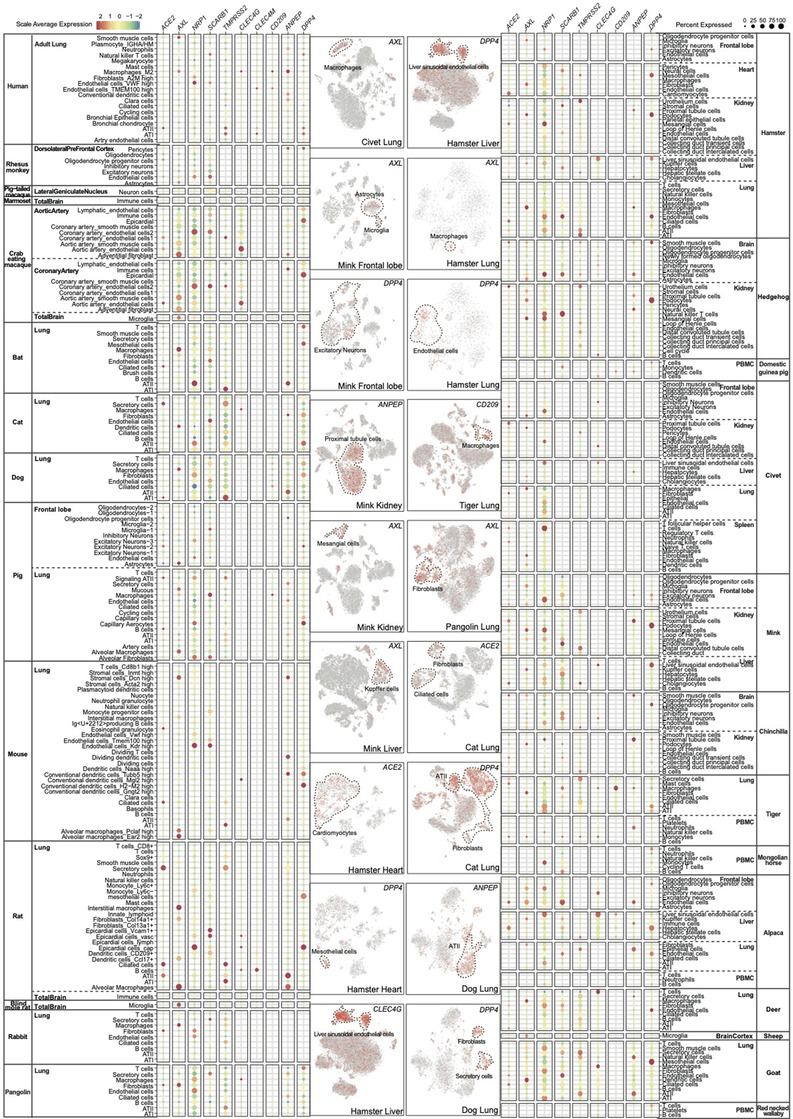
Dot plot showing the expression profiles for the entry factors and cofactors of coronaviruses. The expressions of 10 receptors for coronaviruses were screened in tissues of 27 species. Colour saturation reflects the scaled average expression, while dot size indicates the percentage of cells of each cell type expressing the receptor. The feature plots are displayed to show the specific expression of virus receptors in distinct cell types.

Coronaviruses are among the most common pathogens causing respiratory illnesses in humans.^16^, ^17^, ^18^ We focused our attention on these viruses because of their roles in epidemics and pandemics. We first screened the expression patterns of several SARS‐CoV‐2 entry factors and cofactors in the tissues of 27 species (alpaca, mink, chinchilla, domestic guinea pig, hedgehog, horse, red‐necked wallaby, human,^19^ rhesus monkey,^20^ crab‐eating macaque,^21^, ^22^ pig‐tailed macaque [https://portal.brain‐map.org/], marmoset,^22^ pig,^14^, ^23^ hamster,^15^ mouse,^24^ blind mole rat,^22^ rat,^13^, ^22^ rabbit,^15^ cat,^15^ tiger,^15^ civet,^25^ dog,^15^ pangolin,^15^ goat,^15^ deer,^15^ sheep^22^ and bat^26^). Angiotensin‐converting enzyme 2 (*ACE2*) and the tyrosine‐protein kinase receptor UFO (*AXL*) are the cellular receptors responsible for SARS‐CoV‐2 infection,^8^, ^27^ while trans‐membrane serine protease (*TMPRSS2)*, Neuropilin‐1 (*NRP1*) and the high‐density lipoprotein scavenger receptor B type 1 (*SCARB1*) are cofactors promoting the ACE2‐dependent entry of SARS‐CoV‐2.^28^, ^29^, ^30^ Besides, *ACE2* is also a shared receptor for SARS‐CoV and HCoV‐NL63. Co‐expressions of *ACE2* and its cofactors were identified in the tissues of several species (Figure 2, Table S4), including the brain smooth muscle cells and kidney urothelium cells of hedgehog, the kidney distal convoluted tubule cells and liver cholangiocytes of mink, multiple cell types of hamster (the cardiomyocytes of heart, distal convoluted tubule cells, collecting duct transient cells and collecting duct intercalated cells of kidney, sinusoidal endothelial cells, Kupffer cells and hepatocytes of liver, mesothelial cells, ciliated cells, ATI and ATII cells of lung), the kidney B cells of chinchilla, the secretory cells and ciliated cells in the lung of tiger, the ATII cells in the lung of human, the fibroblasts, ATI and ATII cells in the lung of pangolin, the secretory cells and ciliated cells in the lung of bat, the secretory cell and ciliated cells in the lung of cat, ATI cells in the lung of dog, several lung cells of pig (signalling ATII, B cells, ATI and ATII cells), ciliated cells in the lung of mouse, secretory cells, ciliated cells and ATII cells in the lung of rat, ATII and ATI cells in the lung of deer, ciliated cells in the lung of goat. *ACE2* was mostly detected in less than 25% of the cells of a single cell types, which was lower than *TMPRSS2* and the other receptors for SARS‐CoV‐2. *AXL, NRP1* and *SCARB1* were detected at high levels in the tissues of 21 animals, including hedgehog (brain and kidney), alpaca (frontal lobe, liver and lung), mink (frontal lobe, kidney and liver), hamster (heart, kidney, liver and lung), chinchilla (brain and kidney), tiger (lung and PBMC), human (lung), pangolin (lung), bat (lung), cat (lung), dog (lung), pig (frontal lobe and lung), mouse (lung), rat (lung), blind mole rat (brain), deer (lung), sheep (brain cortex), goat (lung), rabbit (lung), rhesus monkey (dorsolateral prefrontal cortex) and crab‐eating macaque (aortic artery, coronary artery and total brain). The close relative of SARS‐CoV‐2, SARS‐CoV, utilises ACE2, CD209((DC‐SIGN), CLEC4M (DC‐SIGNR) and CLEC4G (LSECtin) for cellular entry.^9^, ^31^, ^32^, ^33^ Among the tissue samples investigated, *CD209* was only detected in the liver of alpaca, the lungs of tiger, human, dog and pig and in the PBMC of domestic guinea pigs. The expression of *CLEC4M* was only observed in a very small fraction of cells in the lungs of human and rat. *CLEC4G* had a higher expression level than *CD209* and *CLEC4M*, especially in alpaca (frontal lobe and liver), hamster (liver), deer (lung) and goat (lung). The receptor for MERS‐CoV, *DPP4*, was widely detected in the tissues examined, for example, in the lungs of alpaca, hamster, human, tiger, pangolin, bat, cat, dog, mouse, rat, deer and goat. Aminopeptidase N (ANPEP) was a receptor for HCoV‐229E,^34^ whose expression was found in the lungs of multiple species in our study, including alpaca, hamster, tiger, human, pangolin, bat, cat, dog, pig, mouse, rat and goat. These results showed that the receptors of coronaviruses were widely expressed by mammalian species.

### 2.3 Screening of receptors for other respiratory viruses in the lung tissues of 18 animal species

Besides coronaviruses, we also screened for the receptor distribution of other viruses causing respiratory infection. Here, we selected 27 viral receptors corresponding to seven viral families (*Adenoviridae, Hantaviridae, Paramyxoviridae, Parvoviridae, Picornaviridae, Pneumoviridae* and *Reoviridae*) and screened their expression patterns in the lungs of 18 species (human, pig, mouse, rat, hamster, cat, dog, tiger, civet, rabbit, alpaca, deer, goat, bat, pangolin, duck,^15^ pigeon^15^ and lizard^15^) (Figure S1). *RPSA*, a receptor for a group of parvoviruses including adeno‐associated virus 2/3/8/9, was widely expressed by different cell types in the lung of human (Clara cells), mouse (dendritic cells, macrophages, B cells, ATII, endothelial cells, ciliated cells, etc.), rat (monocytes, dendritic cells, macrophages, B cells, T cells, ATI, ATII, fibroblasts, epithelial cells, ciliated cells, etc.), hamster (secretory cells), tiger (ciliated cells, ATI, ATII, secretory cells, macrophages, endothelial cells, fibroblasts and mast cells) and pigeon (ciliated cells) (Figure S1, Table S4). Besides the above cell types, *RPSA* was also detected by other cells in various species though with lower expression levels and was generally enriched in immune cells such as macrophages, dendritic cells, B cells and T cells. *CDHR3*, the receptor for rhinovirus C of the *Picornaviridae*, was mainly expressed by lung ciliated cells, although sporadic expressions by other cell types were also observed (Figure S1, Table S4). Integrins are a family of transmembrane receptors which could facilitate both cell‐cell and cell‐extracellular matrix adhesion. They are commonly used as viral receptors for a variety of non‐enveloped and enveloped viruses.^35^ *ITGA5*, a receptor for human parvovirus B19, adeno‐associated virus‐2 (*Parvoviridae*) and human metapneumovirus (*Pneumoviridae*), was found to be mainly enriched in the endothelial cells of multiple animals including the dog, tiger, cat, bat, pig, civet, alpaca, mouse and lizard (Figure S1). High expression of *ITGA5* was also observed in the macrophages of rat, tiger and rabbit*. ITGAV*, a receptor of human mastadenovirus C (*Adenoviridae*) and human parechovirus 1 (*Picornaviridae*), was commonly expressed by different cell types, including secretory cells, monocytes, mesothelial cells, macrophages, fibroblasts, epithelial, endothelial cells, dendritic cells, B cells, ATII and ATI of different species. In contrast, the expression levels of *ITGB3* (the receptor of hantaan orthohantavirus of *Hantaviridae* and human parechovirus 1 of *Picornaviridae*) were much lower and were mainly observed in the monocytes and innate lymphocytes of rat, and the fibroblasts of dog. *ITGB5*, another receptor of human mastadenovirus C, was commonly observed in fibroblasts (rat, civet, dog, hamster, pangolin and tiger) with some expressions in endothelial cells, ciliated cells, ATI, ATII and macrophages. *CXADR* was a receptor shared by multiple adenoviruses, which was mainly detected in ATI (civet, dog, tiger, cat, rat, mouse, deer, goat, pangolin, rabbit, alpaca and pig), ciliated cells (civet, mouse, rabbit, rat, pangolin, bat, pig and human) and ATII (tiger, cat, deer and pig). *EFNB2* and *EFNB3* are both receptors of Henipaviruses (*Paramyxoviridae*). *EFNB2* was especially enriched in the endothelial cells of 16 species, including deer, goat, duck, civet, tiger, cat, hamster, pigeon, dog, lizard, alpaca, rabbit, pangolin, bat, human and mouse, while *EFNB3* was only sporadically detected in the lung cells of some species. CD209, CD46, NECTIN4 and SLAMF1 are receptors of measles morbilliviruses of the *Paramyxoviridae*. Only *CD46* displayed moderate to high expression levels in the lung cells of some species, such as ciliated cells (hamster, alpaca, rat and bat) and secretory cells (pangolin, hamster and rat).

### 2.4 Screening of viral receptors in the PBMC data set of 27 species covering mammals, birds, reptiles and fish

**FIGURE 3:**
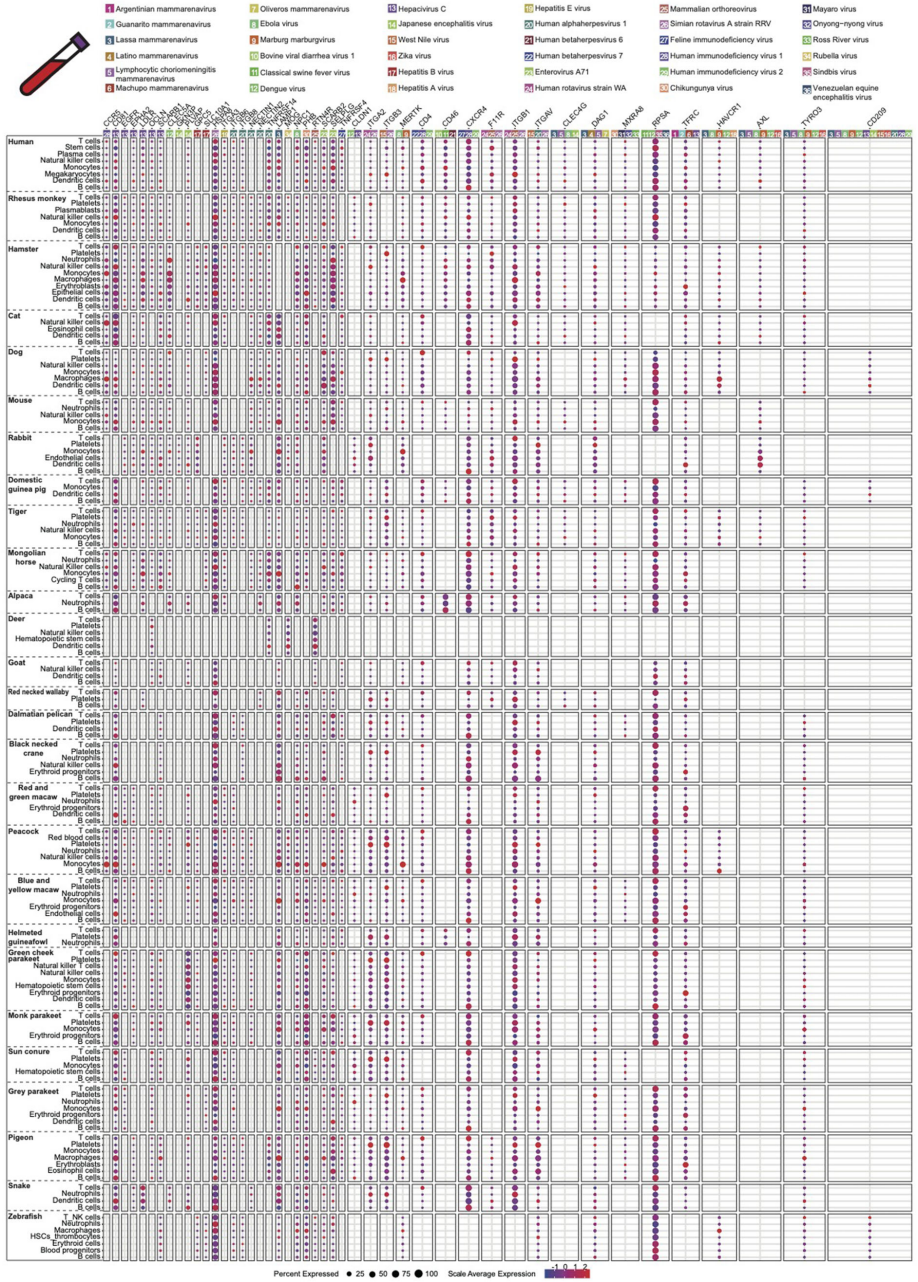
Cross‐species screening of viral receptor expressions in the PBMC data sets. The expressions of 46 associated viral entry factors in the PBMC of 27 species were screened. Colour saturation of dots reflects the scaled average expression, while dot size indicates the percentage of cells of each cell type expressing the receptor. The corresponding viruses for each entry factor are labelled with different colours and numbers, with virus‐specific entry factors on the left side and virus‐shared entry factors on the right side.

We further explored the putative target cells of viruses capable of causing blood infections (Figure 3, Table S4), based on 46 associated viral entry factors belonging to nine virus families (*Arenaviridae, Filoviridae, Flaviviridae, Hepadnaviridae, Herpesviridae, Picornaviridae, Reoviridae, Retroviridae* and *Togaviridae*) using the PBMC data sets of 27 species, which contained the self‐produced dataset of 16 species and the public dataset of 11 species (human,^36^ monkey,^37^ hamster,^38^ cat,^38^ dog,^38^ mouse,^39^ rabbit,^38^ deer,^38^ goat,^38^ pigeon^38^ and zebrafish^40^) (Figure 3). The viruses investigated covered common blood‐borne viruses such as human immunodeficiency virus (HIV, *Retroviridae*), hepatitis B virus (HBV, *Hepadnaviridae*) and Hepacivirus C (HCV, *Flaviviridae*), viruses that can cause haemorrhagic fever (*Arenaviridae*, *Filoviridae*, *Flaviviridae* and *Togaviridae*) and others that can cause hepatitis, such as hepatitis A virus (*Picornaviridae*) and hepatitis E virus (*Herpesviridae*). The known receptors for HIVs included CXCR4, CD4CCR5 and CD209. While *CCR5* and *CD209* were only sporadically detected in certain species, *CXCR4* was abundantly expressed by B cells of mammals (human, hamster, domestic guinea pig, cat, tiger and rabbit), birds (black necked crane, blue and yellow macaw, dalmatian pelican, green cheek parakeet, grey parakeet, monk parakeet, peacock, pigeon, red and green macaw, sun conure) and snake, and by T cells of at least 13 species (human, alpaca, dog, goat, red necked wallaby, horse, dalmatian pelican, peacock, green cheek parakeet, monk parakeet, pigeon, sun conure and snake). CD4 was used as a receptor for both HIVs and human betaherpesvirus 7. Interestingly, while CD4 was found in T cells in most of the studied animals, it was mostly prevalent in dendritic cells and monocytes in humans. Multiple blood cell types displayed low levels of *NPC1* (a receptor of Ebolavirus), including B cells, dendritic cells, macrophages, monocytes, neutrophils, T cells, etc., depending on the species. AXL, TYRO3, MERTK, CLEC4G, CD209 and HAVCR1 are the main receptors for Ebola virus and Marburg virus. Very low and sporadic expressions of *CLEC4G*, *CD209* and *HAVCR1* were identified in the PBMC data sets. High expression of *AXL* was observed in the dendritic and endothelial cells of rabbit. The arenaviruses shared some of the receptors with filoviruses, including *AXL, CLEC4G, CD209, HAVCR1* and *TYRO3*. Besides these genes, the arenaviruses also use DAG1, LAMP1 and TFRC as receptors. *DAG1* exhibited lower expression in different cell types of various animal species, such as B cells (mouse, tiger, red necked wallaby, horse, alpaca, snake and zebrafish), dendritic cells (hamster, domestic guinea pig and snake), natural killer cells (hamster, monkey, horse, cat, tiger, black necked crane, green cheek parakeet, zebrafish), platelets (dog, rabbit, grey parakeet, blue and yellow macaw, red and green macaw, black necked crane, sun conure, green cheek parakeet, pigeon) and T cells (horse, red necked wallaby, green cheek parakeet, dalmatian pelican, helmeted guineafowl). *TFRC* was detected in different cell types in different species, with enrichment in the B cells, dendritic cells, monocytes and plasma cells in the human PBMC data set. HCV (*Flaviviridae*) has several receptors, including CLDN1, EGFR, LDLR, TFRC, EPHA2, CD81, OCLN, SCARB1 and CD209. Among these, *CD81* displayed higher expressions than the other receptors. For HBV of the *Hepadnaviridae*, the viral receptor gene (*SLC10A1* and *GPC5*) expression was barely detected. For *Herpesviridae*, CD209, CD4, CD46, ITGA3, ITGAV, ITGB6, ITGB8, NECTIN1, NECTIN2 and TNFRSF14 were recognised as receptors. However, only *CD4, ITGAV* and *TNFRSF14* displayed sporadic high expression in some species, such as human, hamster and dog. For Enterovirus A71 of the *Picornaviridae*, its receptors (*SCARB2* and *SELPLG*) were distributed in different cell types including T cells, monocytes, dendritic cells and macrophages of different species. Dogs showed higher expression of all the two receptors, while only *SELPLG* had higher expression in human and hamster PBMC. For *Reoviridae*, *HSPA8* was abundantly expressed by B cells, natural killer cells, neutrophil, T cells of 25 species in this study. *ITGA2, ITGB1* and *ITGB3* were commonly detected in platelets of mammals and birds, while moderate expression of *F11R* was only observed in human, hamster, rabbit and tiger. The togaviruses use MOG, MXRA8, PHB and RPSA as receptors, and only *RPSA* and *PHB* had high expression in the animal PBMC data set, especially *RPSA*, which also serve as a receptor for flaviviruses.

### 2.5 Screening of entry factors for neurotropic viruses in the brain tissues of mammals and reptiles

**FIGURE 4:**
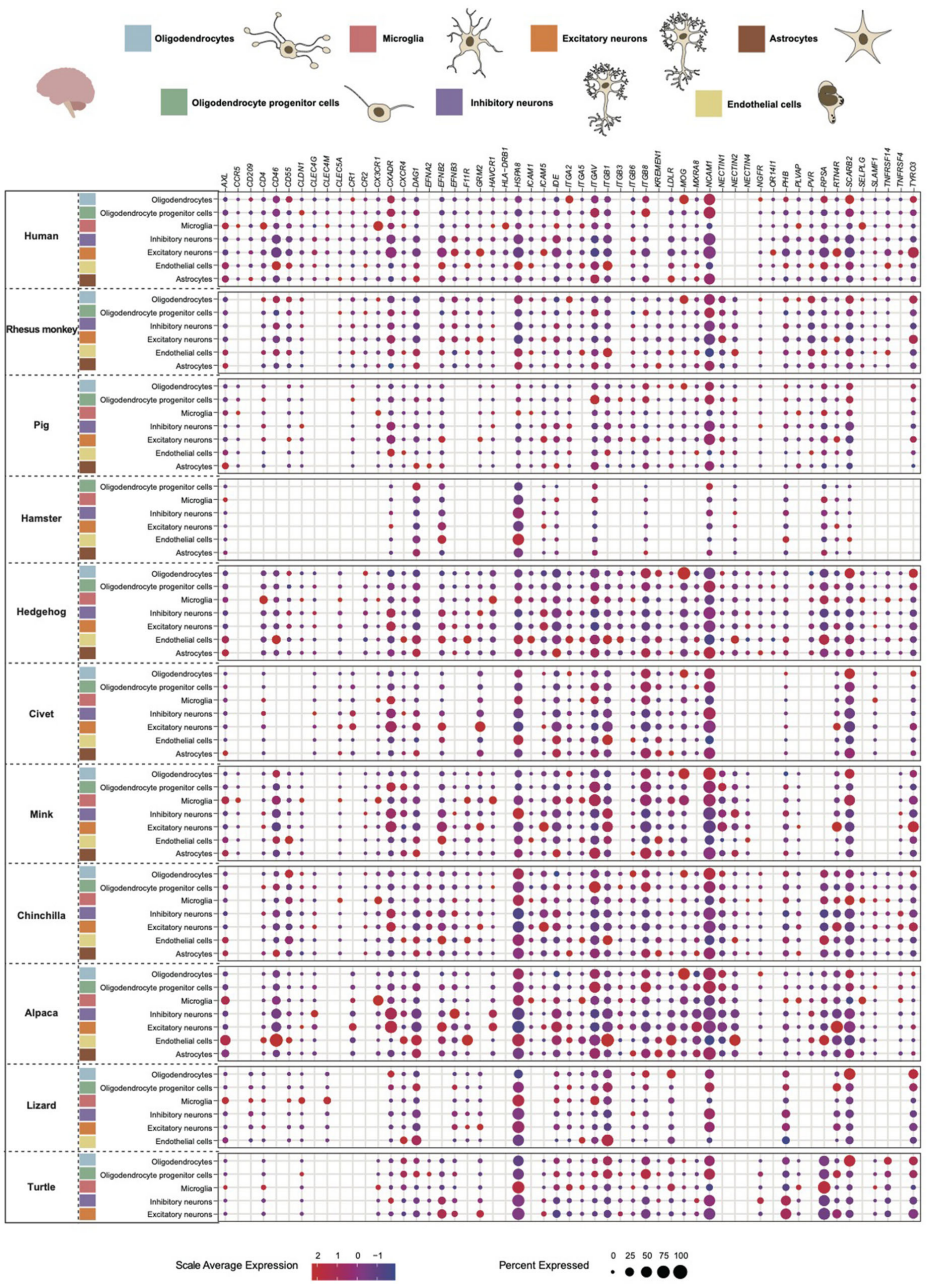
Dot plot showing cross‐species screening of neurotropic virus putative target cells in brain tissues. The expressions of 55 receptors for neurotropic viruses from 11 viral families (*Arenaviridae, Flaviviridae, Herpesviridae, Paramyxoviridae, Phenuiviridae, Picornaviridae, Pneumoviridae, Reoviridae, Retroviridae, Rhabdoviridae* and *Togaviridae*) in the brain tissues of 11 species covering 9 mammals (human, monkey, pig, hamster, hedgehog, civet, mink, chinchilla and alpaca) and 2 reptiles (lizard and turtle) were screened. Different cell types are annotated with different colours based on scRNA‐seq data. Colour saturation of dots reflects the scaled average expression, while dot size indicates the percentage of cells of each cell type expressing the receptor.

Neurological complications can be caused by neuroinvasive viruses including polioviruses, rabies viruses, flaviviruses, herpes simplex viruses and measles. In addition to the neuroinvasive viruses, a variety of other viruses such as coronaviruses and influenza viruses, are also capable of infecting the brain tissues under certain circumstances. To explore the putative target cells and hosts of the neurotropic viruses, we systematically screened the expression of 55 viral receptors from 11 viral families (*Arenaviridae, Flaviviridae, Herpesviridae, Paramyxoviridae, Phenuiviridae, Picornaviridae, Pneumoviridae, Reoviridae, Retroviridae, Rhabdoviridae* and *Togaviridae*) in the brain tissues of 11 species covering 9 mammals (human, monkey, pig, hamster, hedgehog, civet, mink, chinchilla and alpaca) and 2 reptiles (lizard and turtle). *SCARB2*, a receptor for coxsackievirus A16 and enterovirus A71, was highly expressed by oligodendrocytes in the brain (expression percentage > 15%) of human,^41^ monkey, hedgehog, civet, mink, alpaca, lizard^12^ and turtle^12^ (Figure 4, Table S4). In addition to oligodendrocytes, *SCARB2* was also found to be expressed by the endothelial cells of monkey aUnd hedgehog, microglia of chinchilla and mink, and oligodendrocyte progenitor cells of pig and alpaca. NCAM1 is a receptor for rabies lyssavirus of *Rhabdoviridae*. Conserved expression of *NCAM1* was observed in oligodendrocytes of human, pig, civet, mink, chinchilla and alpaca and oligodendrocyte progenitor cells of all species investigated. Sporadic expressions of *NCAM1* by astrocytes, inhibitory neurons and microglia were also detected. These observations are consistent with a wide host range of rabies viruses. *HSPA8*, a receptor for Simian rotavirus A strain RRV of *Reoviridae*, was widely expressed (expression percentage > 50%) by diverse cell types in the brain of hamster (endothelial cells and inhibitory neurons), mink (inhibitory neurons), chinchilla (oligodendrocytes and microglia), alpaca (endothelial cells, oligodendrocytes and microglia), lizard (excitatory neurons and microglia) and turtle (microglia). *MOG* is a receptor for the rubella virus of *Togaviridae* and was specifically expressed by oligodendrocytes in the brain of mammals including human, monkey, hedgehog, civet, mink, chinchilla and alpaca. *ITGB8*, a receptor for human alphaherpesvirus 1 of *Herpesviridae*, was mainly expressed by oligodendrocytes (hedgehog, civet, mink, chinchilla, alpaca and lizard), oligodendrocyte progenitor cells (human, civet, chinchilla, alpaca and turtle) and astrocytes (hedgehog, civet, mink and chinchilla).

### 2.6 Conservation of brain connectomes

**FIGURE 5:**
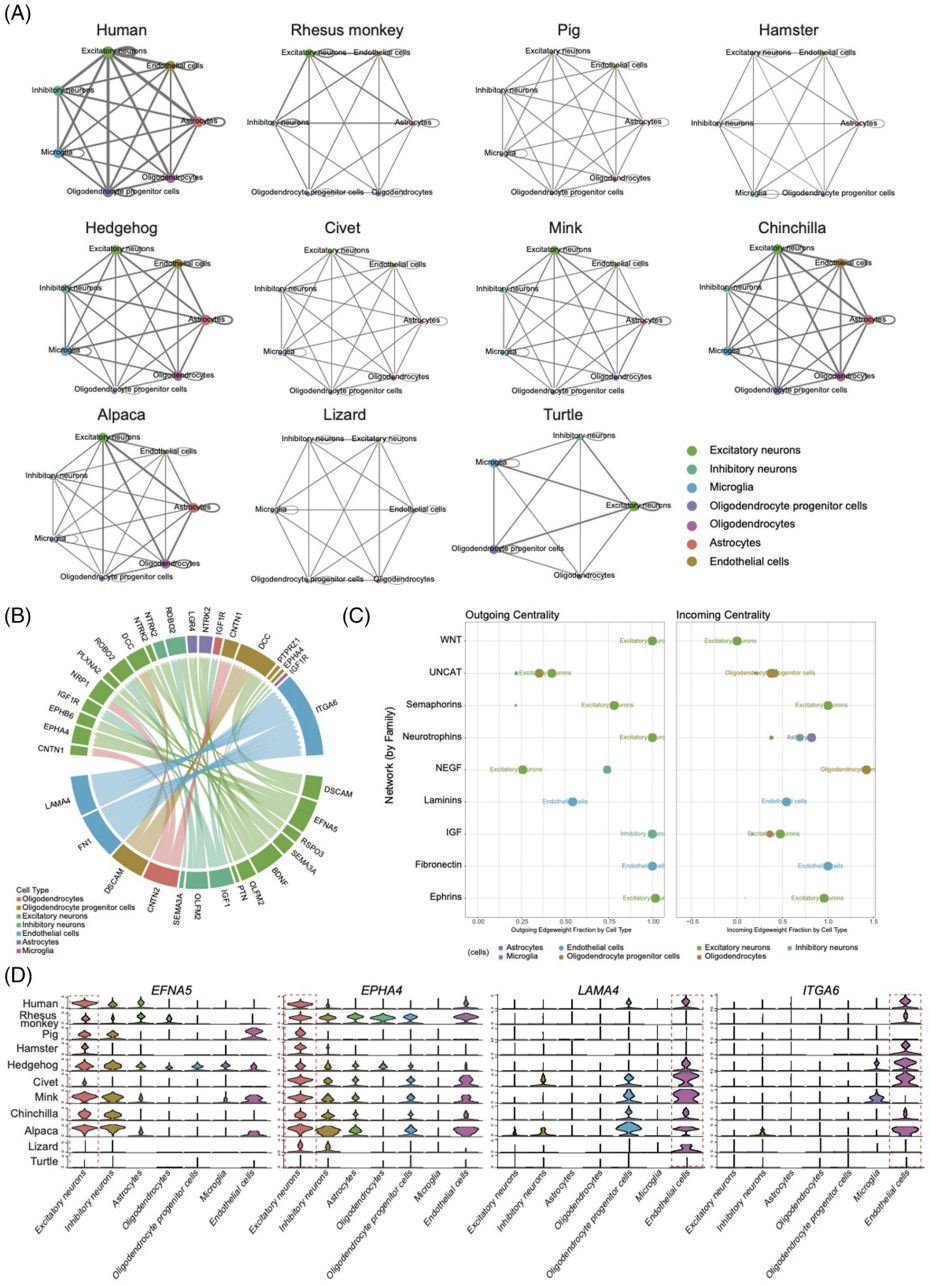
Conservation and divergence of brain cell connectomes. (A) A ligand‐receptor mediated interaction network for brain cells within 11 species (human, monkey, pig, hamster, hedgehog, civet, mink, chinchilla, alpaca, lizard and turtle). Different cell types are annotated with coloured nodes across species, of which the size is proportional to the sum of ligand‐receptor pairs between a cell type with all other cell types. The edge thickness is proportional to the sum of ligand‐receptor pairs between two cell types. (B) Circos plot displayed the cell‐cell interactions conserved among all 11 species. Receptors and ligands occupy the upper and lower semicircle respectively. (C) Network centrality analysis shows the source weight and hub score of various cell types in indicated networks across 11 species. (D) Violin plots showing the conserved expression of *ephrin A5* (*EFNA5*) and *EPH receptor A4* (*EPHA4*) in excitatory neurons, and laminin subunit alpha‐4 (*LAMA4*) and integrin alpha‐6 (*ITGA6*) in endothelial cells across 11 species.

In addition to studying the cell‐virus interactions, our single‐cell resource can be used to investigate the divergence and conservation of cross‐talks between cells throughout evolution. To identify putative cellular communications, a ligand‐receptor mediated interaction network was constructed for brain cells within 11 species covering 9 mammals (human, monkey, pig, hamster, hedgehog, civet, mink, chinchilla and alpaca) and 2 reptiles (lizard and turtle) (Figure 5A, Table S5). A close interaction between excitatory neurons and astrocytes was observed in human, which was conserved in animals including monkey, hedgehog, mink, chinchilla and alpaca. Whereas the close connection between excitatory neurons and oligodendrocyte progenitor cells was specific to human, indicating significant heterogeneity in the cross‐talk of brain cells in humans. We next identified pan‐conserved cellular connectivity, which may correspond to ancient signalling vectors inherited from common ancestors of mammals and reptiles. In total, we detected 291 pairs of cell‐cell interactions conserved among all the 11 species, 44 pairs of which were mediated through the brain derived neurotrophic factor (BDNF)‐neurotrophic receptor tyrosine kinase 2 (NTRK2) signalling (Figure 5B, Table S5). The BDNF‐NTRK2 signalling plays an important role in neuronal differentiation, maintenance and plasticity.^42^ Here, our results demonstrated that the BDNF‐NTRK2 signalling was transduced from excitatory neurons into diverse cell types, including astrocytes, excitatory neurons, inhibitory neurons, microglia and oligodendrocytes. This indicates that excitatory neurons may have a core regulatory role in brain cell connectomes across these 11 species.

In addition to pan‐conserved cellular cross‐talk, we explored which cell types were predominant in cell‐cell cross‐talk. In total, we found excitatory neurons appended in most families either outgoing centrality or incoming centrality, and endothelial cells followed closely (Figure 5C). Excitatory neurons received conserved cellular interactions transduced from excitatory and inhibitory neurons, oligodendrocytes progenitor cells and oligodendrocytes, a large portion of which were mediated through inhibitory neurons. Notably, we found that the olfactomedin 2 (OLFM2)‐roundabout guidance receptor 2 (ROBO2) signalling could be transduced from inhibitory neurons to excitatory neurons, and vice versa, which has not been observed in neurons before. Intriguingly, we found that the identified pan‐conserved cellular cross‐talk associated with endothelial cells was entirely mediated between endothelial cells. This indicates that cellular cross‐talk between endothelial cells is quite conservative, whereas the interactions between endothelial cells with other types of cells may be species‐specific.

The expression of known ligands and receptors was examined to confirm the dominant role of excitatory neurons and endothelial cells across species. For example, ephrin A5 (EFNA5) and EPH receptor A4 (EPHA4) as the ligand and receptor of the ephrin family were commonly expressed in excitatory neurons. The ligand and receptor of laminins family, laminin subunit alpha‐4 (LAMA4) and integrin alpha‐6 (ITGA6) were also highly expressed in endothelial cells of most species (Figure 5D). In conclusion, this study systematically revealed both highly conserved and lineage specific cell‐to‐cell signalling within the vertebrate brain for the first time.

### 2.7 Conservation of brain regulomes

**FIGURE 6:**
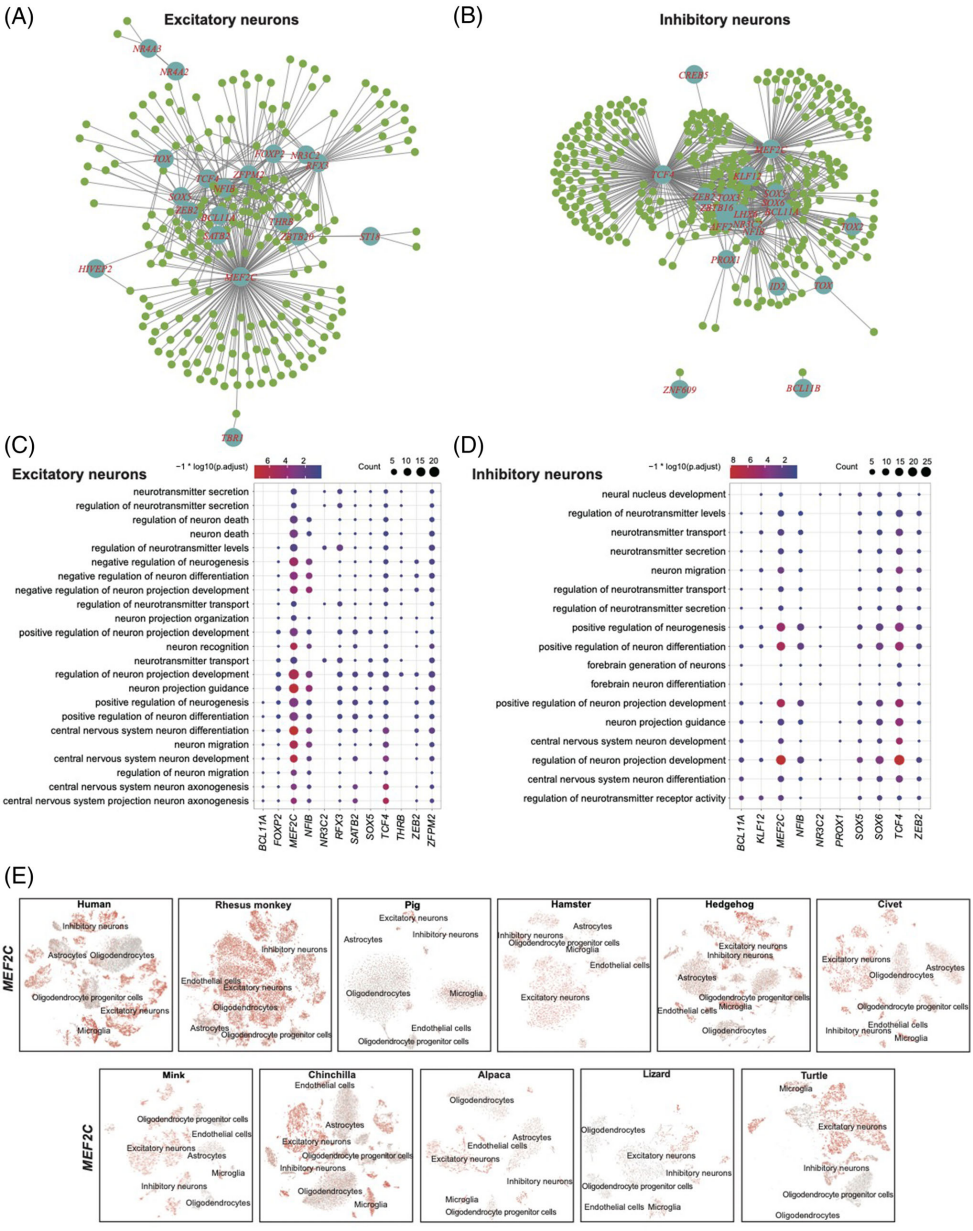
Conservation and divergence of brain cell regulomes. Gene regulatory network of the conserved TFs and target genes detected across 11 species in excitatory neurons (A) and inhibitory neurons (B). Blue nodes with labels represent the TFs and the smaller green nodes represent the target genes. GO enrichment of the target genes of key TFs in excitatory neurons (C) and inhibitory neurons (D). Colour saturation of dots reflects the significance level of enrichment analysis, while dot size indicates the count of target genes classified in each GO term. (E) Feature plots displaying the conserved expression of MEF2C in excitatory neurons and inhibitory neurons across 11 species.

To reveal the regulatory mechanisms underlying neural cells from the perspective of evolutionary biology, we predicted the regulomes in neural cells for all the 11 species, resulting in a total of 170 702 TF‐target interactions in excitatory neurons and 383 544 TF‐target interactions in inhibitory neurons (GENIE3 score > 0.01) conserved in at least 4 species. TF genes and target genes expressed in 11 species ranged from 461 in excitatory neurons cells to 678 in inhibitory neurons cells (Figure 6A and B, Tables S6 and S7). Notably, several well‐known regulators for excitatory neurons were active in the genetic regulatory network of the corresponding cell types, consistent with their expected regulatory functions. Additionally, we found a variety of regulatory circuits that were highly conserved among multiple species. Briefly, the interactions between MEF2C its target genes (*EPHA4*, *PEX5L*, *CNTN3* and *PHACTR1*), were conserved in excitatory neurons cells of at least 8 species. In inhibitory neurons, the regulatory relationships between MEF2C and its target genes (*CACNA2D3*, *MMP16*, *PPARGC1A*, *RELN*, *SOX5*, *SOX6*, *ATRNL1* and *KCNC2*) were conserved in more than 8 species. The transcription factor MEF2C is crucial for normal neuronal development in humans and monkey.^43^, ^44^ Consistent with this, our analysis indicated a strongly conserved expression of MEF2C and a conserved core regulatory role of MEF2C across all species.

The regulatory functions of core transcription factors were inferred based on the enriched GO terms of their predicted target genes. The target genes of MEF2C were enriched in GO terms associated with positive regulation of neurogenesis, positive regulation of neuron differentiation and regulation of neuron projection development in either excitatory neurons or inhibitory neurons (Figure 6C and D, Table S8). The conserved and key regulatory roles of MEF2C in both excitatory neurons and inhibitory neurons across species suggest that MEF2C may participate in the maintenance of the balance of cerebral excitatory and inhibitory synapses in a species‐conserved manner. Whereas the different conserved target genes of MEF2C in excitatory neurons and inhibitory neurons indicate the diverse regulatory functions of MEF2C in different cell types. We also found that MEF2C was commonly expressed in all the studied species (Figure 6E). Overall, our study systematically identified a conserved core regulatory program for brain cells, including both well‐recognised and novel regulators (Figure 6).

## 3 DISCUSSION

Overall, this study characterised the expression of receptors used by a variety of viruses in 75 tissues from 44 species from mammals, birds, reptiles and fishes. The resources provided by this study may assist the exploration of tissue and host tropism of corresponding viruses, direct experimental design and guide the choice of appropriate animal infection models.

**FIGURE 7:**
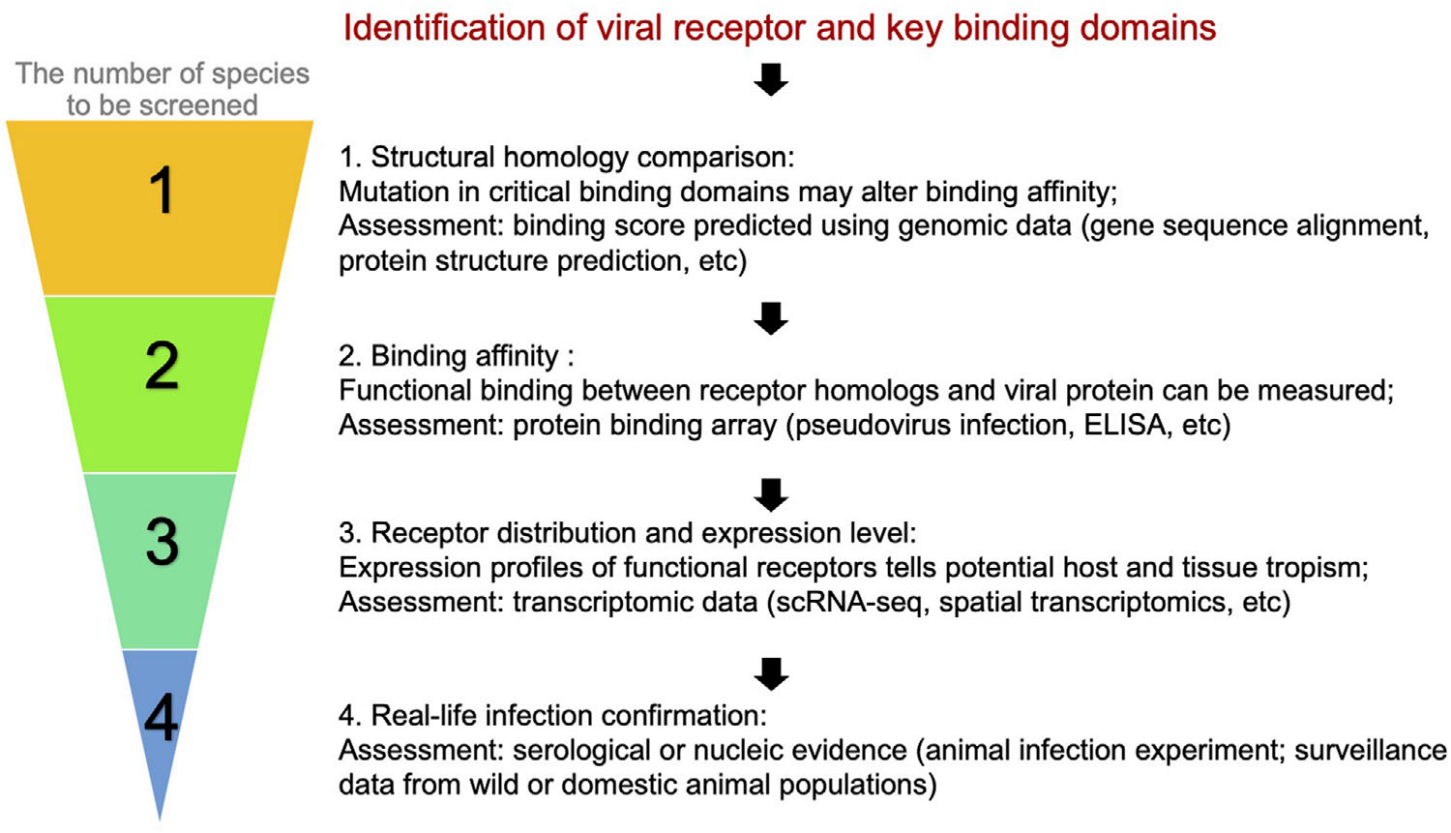
An integrative multi‐omics workflow to identify the host range of novel pathogens.

Defining the pathogen's key cellular receptors is the first step in determining its potential host range. With the key binding sites unravelled, genomic data can be used to compare the structural similarity of the receptor homologs between different species. If the structural similarity is high enough to support potential interaction between the virus and an animal species, we can further move to construct the protein sequences of the viral binding domains and the receptor homologs, then assess their binding affinity using experimental methods such as pseudovirus infection assay, flow cytometry, western blot and ELISA. With proper positive and negative control, we would be able to know the receptor of which species may support virus entry. However, different viruses vary in their infection routes and tissue tropisms, a further understanding of the viral receptor distribution and expression levels in different organs would help reveal the anatomical sites potentially infected or diseased by the virus. Such analysis can be readily supported by the scRNA‐seq data as provided in this study. Further confirmation studies using in vitro and/or in vivo infection experiments, or field surveillance data originated from serology test or nucleotide detection can finally confirm the susceptibility of a certain animal species to our target pathogen. Following this rationale, we can narrow down the suspect host list step by step (Figure 7). From the very beginning of the COVID‐19 pandemic, scientists have already tried to pinpoint the animal host range of SARS‐CoV‐2 using some of the above methods.^8^, ^45^, ^46^, ^47^, ^48^, ^49^, ^50^, ^51^, ^52^ Upon the discovery of the etiological agent of COVID‐19, ACE2 has been recognised as the receptor of SARS‐CoV‐2, and the critical sites composing the binding surface between the viral spike protein and ACE2 was soon identified.^8^, ^45^, ^46^ Later, a comparative study on the conservation of ACE2 in different animal species was carried out using the ACE2 sequence data.^47^ The binding affinities between a bunch of ACE2 orthologs and the functional domains of SARS‐CoV‐2 spike protein were also evaluated.^48^ To reveal the tissue tropism of SARS‐CoV‐2 in the human body, the expression landscape of ACE2 and its cofactor TMPRSS2 was characterised by several groups.^49^, ^50^, ^51^, ^52^ Similarly, our group has utilised scRNA‐seq data to explore the host range and tissue tropism of SARS‐CoV‐2.^15^ It's noteworthy that when ACE2 in white‐tailed deer was reported to exhibit high binding scores to SARS‐CoV‐2 spike protein,^47^ subsequent serology surveillance in wild individuals and laboratory infection experiments further supported this species as a permissive host of SARS‐CoV‐2,^53^, ^54^ extending our understanding of SARS‐CoV‐2 behaviour in the real‐world. However, it should be noted that most of the related research work was individually conducted by different groups separately and perhaps simultaneously, which didn't help to reduce the suspect list in a stepwise manner and each step remains resource expensive. What we called for, is the close interdisciplinary collaborations between bioinformatic and bench scientists to realise the OneHealth concept, through effective integration of multi‐omics data including genomic, transcriptomic, proteomic and surveillance data. The related technologies should be progressively combined and operated within an organised network, so that our scientific questions can be solved more systematically, effectively and economically. Despite the positive outlook, we must recognise that much work remained to be done to fulfil this framework. First, the genome sequences of a lot of species are still lacking. For example, bats are reservoir hosts for abundant viruses, yet less than 100 of the over 1400 bat species have their genomes sequenced^55^, ^56^, ^57^ (https://bat1k.com/progress/), hindering our investigation on the role of these animals in virus maintenance and transmission. Second, a virus may employ not just one cellular surface protein as its binding target, and the involvement of alternative receptors inevitably complicates our judgement on viral infectivity and pathogenesis. Built on previous experience of SARS‐CoV, Zhou et al.^8^, ^30^ soon confirmed ACE2 as an entry factor for SARS‐CoV‐2, with TMPRSS2 as its cofactor. However, the common pneumonia cases caused by SARS‐CoV‐2 seemed to contradict the low co‐expression of ACE2 and TMPRSS2 in human lung cells, as revealed in this and previous reports.^49^, ^58^ Later, other receptors and entry cofactors of SARS‐CoV‐2 were uncovered, including *AXL*, *NRP1*, *SCARB1* etc.,^27^, ^28^, ^29^ which may explain its infection in tissues with low ACE2 expression. This kind of hidden information may be responsible for the incongruence between binding affinity results and field observations. However, it remains hard to use scRNA‐seq data to measure how the different entry factors and cofactors contribute to the in vivo viral infection profile, such as the expression threshold for a positive prediction. Furthermore, little is known about the entry factors of many viruses. Third, not all viruses use proteins as their receptors. A prominent example will be influenza A viruses, which utilise sialic acid with different linkages as receptors.^59^ Such post‐translational modifications can't be predicted based on transcriptomic data. Fourth, receptor binding affinity is the mutual effect of viral and host protein. The binding affinity between a defined virus variant and a receptor protein can't fully reflect the interaction behaviour between a new variant and the same receptor. Therefore, the virus binding affinity must be determined separately for different species and even variants within the same species. Fifth, transcriptomic data for many species and tissues remain scarce. Such data may be available for human and classical animal models but not for the thousands of wild species involved in the field of virology, although network centrality studies suggest carnivores, bats and ungulates hold central positions in sharing viruses with other hosts.^3^, ^4^ The process to generate their transcriptome data, especially scRNA‐seq data, would be time and resource consuming. However, with the advancement of technology and accumulation of genomic and transcriptomic data, we should be aware of the potential benefits that these massive multi‐omics data could bring to virology study and build up the knowledge system with an appropriate design. Right now, the research rationale we proposed may appear implausible and distant, but one day, scientists will be able to quickly predict the potential host and tissue tropism of novel zoonotic pathogens based on these resources before turning to animal infection experiments and field investigation.

To have a conclusive view of the cell tropism of viruses, we combined all the receptor expressions obtained by this study and used a threshold of > 15% expression and > 0.5 scaled expression level to filter out receptors with obvious expressions. Nine receptors (CDHR3, CXADR, CXCR4, EFNB2, HSPA8, ITGA2, ITGB1, ITGB3 and RPSA) displayed cell‐specific expression in at least 10 species. In the lung, CDHR3 was enriched in the ciliated cells in 15 species, serving as a receptor of rhinovirus C viruses, indicating that ciliated cells in the respiratory tract may be the main target of this virus. CXADR was a receptor for human mastadenoviruses and was mainly expressed by lung ATI cells. EFNB2 was mainly expressed by lung endothelial cells, facilitating the cell entry of Nipah henipavirus. In the blood, B cells, T cells and platelets support the expression of multiple viral receptors. CXCR4, the receptor for HIV‐1, HIV‐2 and feline immunodeficiency virus, was highly expressed in both B and T cells. A similar distribution was also identified for RPSA, which helps the cell binding for various viruses, including classical swine fever virus, dengue virus, Sindbis virus and Venezuelan equine encephalitis virus. HSPA8, a receptor of simian rotavirus A strain RRV, was highly expressed by the T cells of 14 species. The platelets commonly expressed three receptors, including ITGA2 (a receptor for human rotavirus strain WA and simian rotavirus A strain RRV), ITGB1 (a receptor for human rotavirus strain WA, mammalian orthoreovirus and simian rotavirus A strain RRV) and ITGB3 (a receptor for West Nile virus and simian rotavirus A strain RRV). Understanding the distribution of commonly preferred receptors could help explain the potential tissue tropisms of multiple pathogens. The cells expressing receptors may have a higher chance to assist the initiation of viral infection and promote the intra‐host spread of the viruses. Drugs targeting common major viral receptors may facilitate the prevention and treatment of multiple virus infections.

However, there are several limitations in our study and the results should be interpreted cautiously. (i) We were only able to obtain scRNAseq data for limited number of tissues from each animal species in this study, due to difficulty in sampling and experimental failures. A more comprehensive characterisation of the disease‐related organs is needed for the interpretation of clinical symptoms induced by infections of various viruses in the animals. (ii) The dataset of each organ for each species lacked biological and technical replicates. Therefore, we failed to reveal the comprehensive cell type compositions of each tissue, which hindered the cross‐species comparisons on cell types. Our data may also be influenced by sampling bias due to the individual differences in age, gender, health status, etc. To reduce sampling bias, we have tried to use a relatively high filtering threshold to keep only reliable results. (iii) The expression profiles of the receptors were characterised based on the RNA sequencing data, rather than their mature protein form with appropriate post‐translational modifications. The influenza A viruses, as mentioned above, utilise sialic acid receptors and the sialyl linkage types to determine the differential binding specificity of avian and human adapted strains.^59^ Although the transcriptome data could reveal the presence of the proteins associated with the receptors, the exact expression profiles of their receptors could not be stated by this data. Therefore, influenza viruses were not included in this study. (iv) We have only characterised the known receptor genes, other potential alternative receptors of the viruses were not included, but their distribution could also affect the tissue tropism of the viruses. (v) Both the expression profiles and the binding affinity between key cellular receptors and viral proteins are essential for viral entry into the host cells. Because protein products of the same ortholog may have different structures, and mutations in the binding domain may reduce or even abolish its binding ability to the corresponding viral surface proteins. The tissue tropism and host range of the viruses should be confirmed by experiments or epidemiological evidence. (vi) For some animal species lacking high‐resolution genomic data, accurate determination of their gene expression levels remains challenging. The viral receptor screening, connectome and regulome analyses are based on bioinformatic predictions. Therefore, our data needs to be interpreted with caution and awaits further experimental validation.

In addition to investigating the cell‐virus interactions, the datasets generated in this study can provide a comprehensive data source for the deep analysis of cross‐talks between cells and genes. Cell‐cell communication is the main driving factor of cell differentiation and physiological function, which determines tissue/organ development, homeostasis and response to injury. In tissues, cells communicate directly with local neighbours through paracrine signalling and cell‐cell direct contact, or exchange information with remote partners through endocrine signalling. The emergence of single‐cell technology makes it possible to reveal cell‐specific ligand receptor patterns comprehensively. Here, we explored the cellular communications for brain cells within 11 species, and identified the excitatory neurons as the predominant cell type in cell‐cell cross‐talk. The investigation of the regulomes in neural cells for all the 11 species also identified conserved regulators for brain cells. These results suggest that the generated datasets can facilitate the research community for their personalised needs.

## 4 MATERIAL AND METHODS

### 4.1 Ethics statement

This project was approved by the Institutional Review Board on the Ethics Committee of BGI (Approval letter reference number BGI‐NO. BGI‐IRB A20008‐T2). All procedures were conducted according to the guidelines of the Institutional Review Board on the Ethics Committee of BGI.

### 4.2 Materials description

The construction of transcriptome libraries was first performed on 20 species, of which 5 species, alpaca (*Vicugna pacos*), mink (*Neovison vison*), chinchilla (*Chinchilla lanigera*), hedgehog (*Atelerix albiventris*) and hamster (*Mesocricetus auratus*), were performed using 10× Genomics' Chromium and single cell sequencing libraries. As for green cheeked parakeet (*Pyrrhura molinae*), red and green macaw (*Ara chloropterus*), grey parakeet (*Psittacus erithacus*), dalmatian pelican (*Pelecanus crispus*), peacock (*Pavo cristatus*), tiger (*Panthera tigris altaica*), domestic guinea pig (*Cavia porcellus*), alpaca (*Vicugna pacos*), monk parakeet (*Myiopsitta monachus*), helmeted guineafowl (*Numida meleagris*), snake (*Ptyas mucosa*), blue and yellow macaw (*Ara ararauna*), sun conure (*Aratinga solstitialis*), horse (*Equus caballus*), red necked wallaby (*Notamacropus rufogriseus banksianus*) and black necked crane (*Grus nigricollis*), were performed using scRNA‐seq with DNBelab C4 platform. Next, BGI‐seq 500 sequencer was used for the sequencing of transcriptome libraries. In addition to the self‐generated data, we also retrieved single‐cell transcriptome sequencing data from 26 species, civet (*Paguma larvata*), bat (*Rhinolophus sinicus*), blind mole rat (*Nannospalax galili*), cat (*Felis catus*), crab‐eating macaque (*Macaca fascicularis*), deer (*Cervus nippon*), dog (*Canis lupus familiaris*), goat (*Capra aegagrus hircus*), hamster (*Mesocricetus auratus*), human (*Homo sapiens*), marmoset (*Callithrix jacchus*), mouse (*Mus musculus*), pangolin (*Manis javanica*), pig (*Sus scrofa domestica*), pig‐tailed macaque (*Macaca nemestrina*), rabbit (*Oryctolagus cuniculus*), rat (*Rattus norvegicus*), rhesus monkey (*Macaca mulatta*), sheep (*Ovis aries*), tiger (*Panthera tigris altaica*), pigeon (*Columba livia domestica*), zebrafish (*Danio rerio*), lizard (*Pogona vitticeps*, *Anolis carolinensis*), turtle (*Trachemys scripta elegans*), duck (*Anas platyrhynchos domesticus*), from the public papers or databases (Table S1).

### 4.3 Sample treatment

Tissue samples were dissected and rinsed with 1× PBS to remove the surface blood. Next, the processed samples were frozen in liquid nitrogen for about 10 minutes, and then transferred to a liquid nitrogen tank for storage till further use. The whole blood sample of the animal was taken from the adult living animal and then immediately placed in an EDTA anticoagulant tube. Tissue samples and PBMC samples were treated following previous protocols.^15^

### 4.4 Homologs conversion

Homologs were downloaded from BioMart and genes from each species were converted to human homologs to allow cross‐species comparison. In situation of species lacking homologs in Ensembl database, we identified single‐copy orthologs using OrthoFinder v2.3.3 (orthofinder ‐f input_dir ‐t 10 ‐a 10).^60^ The analysis is to perform sequence alignment for the protein FASTA files of two species, and subsequently calculate the orthologous relationship of protein‐coding genes between different species. If a 1: 1 match existed between a non‐human and human gene, the non‐human gene name was converted to the human gene name. And other genes that are not a one‐to‐one match will be filtered out.

### 4.5 Single‐nucleus RNA‐sequencing data processing

Single‐nucleus RNA‐sequencing (snRNA‐seq) data and gene expression matrix were obtained using Cell Ranger 3.0.2 (10× Genomics) and all gene expression matrices were processed with R package Seurat.^61^ Briefly, cells with gene numbers lower than 200 were filtered out. Cells with mitochondrial percentage higher than 10% were discarded as well. Then, ‘LogNormalize’ method was used for data normalisation. ‘FindVariableFeatures’ function implemented in Seurat was utilised to select the top 2000 highly variable genes. Subsequently, data were scaled and principal component analysis (PCA) was conducted using variable genes. Significant principal components (*p*‐value less than .01) were selected for clustering with the shared nearest neighbour (SNN) based method implemented in ‘FindClusters’ with resolution of 1.0. Finally, the clustering result was visualised using t‐distributed stochastic neighbour embedding (t‐SNE) graphs.

### 4.6 Differential expression analysis and cell types annotation

Differentially expressed genes (DEGs) were identified by ‘FindAllMarkers’ function in Seurat. *p* Values were adjusted with Benjaminiand Hochberg (BH) method, and pathways with an adjusted *p* value ≤.05 were considered significantly enriched. DEGs, GO pathways and verified markers were combined to annotate cell types for each data set. First, canonic cell type markers were collected from published literatures. The expression patterns of cell type marker were used to infer cell type identity. DEG gene list was used to perform GO term enrichment analysis using clusterProfiler package^62^ in order to confirm the cell type annotation results.

### 4.7 Public data collection and processing

Public single cell data sets were downloaded from NCBI, GEO, Human Cell Atlas Portal and EBI single cell database. All public data sets were processed using the same criteria according to the previous studies.

### 4.8 Prediction of host tropism and target cells of viruses

All viruses and corresponding receptors were collected from the viral receptor database (http://www.computationalbiology.cn:5000/viralReceptor),^63^ Human Lung Cell Atlas (HLCA) database^64^ or published articles.^30^, ^65^, ^66^ The expression pattern of these receptors was calculated in all cell types of each data set.

### 4.9 Brain cellular communication analysis

Brain data sets from 11 species (*Homo sapiens*, *Macaca mulatta*, *Sus scrofa domestica*, *Mesocricetus auratus*, *Erinaceus europaeus*, *Paguma larvata*, *Neovison vison*, *Chinchilla lanigera*, *Vicugna pacos*, *Pogona vitticeps* and *Trachemys scripta elegans*) were used to perform communication analysis using R package Connectome. We selected the FANTOM5 database^67^ which included all literature‐supported human ligands and receptors. Connectome networks were constructed according to ‘CreateConnectome’ function. We selected conserved cellular interactions in brain data sets from these 11 species based on the following criteria: ligands and receptors expressed in all data sets and the interaction expressed in at least 8 data sets. A Circos plot was used to visualise conserved interactions.

### 4.10 Brain TF‐target interaction analysis

The human TF list was downloaded from the animalTFDB,^68^ and protein coding genes were selected for downstream analysis. We also filtered out the TFs and genes which were expressed in less than 5% of excitatory and inhibitory neurons. For each data set, putative regulatory circuits were predicted by GENIE3 package,^69^ and TF‐target interactions with weight value ≥0.01 were retained.

## CONFLICT OF INTEREST

The authors declare no competing interests.

## Supporting information

Figure S1. Cross‐species screening of respiratory virus target cells in lung tissues. (A) The species‐split dot plot shows the screening of the expression of respiratory virus receptors in all cell populations profiled in lung tissues. Colour saturation of dots reflects the scaled average expression, while dot size indicates the percentage of cells of each cell type expressing the receptor. (B) The specific expression of CDHR3, a receptor for rhinovirus C of Picornaviridae, in ciliated cells across species

Table S1. List of sample information and cell markers in all samples. The metadata of samples used in this study are provided. The markers for cells from tissues of different species are displayed

Table S2. DEGs in cells of different tissues across all species

Table S3. GO terms of DEGs in cells of different tissues across all species

Table S4. Expressions of receptors for coronaviruses and viruses infecting PBMC, brain and lung

Table S5. Brain cellular connectomes across 11 species. All ligand‐receptor pairs expressed in brain cells across 11 species including 9 mammals (human, monkey, pig, hamster, hedgehog, civet, mink, chinchilla and alpaca) and 2 reptiles (lizard and turtle) are listed. Filtered ligand‐receptor pairs conserved in at least 8 species are displayed

Table S6. List of regulomes of excitatory neurons in the brain across 11 species. The TFs and corresponding target genes expressed by excitatory neurons are shown in species including human, monkey, pig, hamster, hedgehog, civet, mink, chinchilla, alpaca, lizard and turtle. The TFs and target genes conserved in excitatory neurons of at least 4 species are also presented here

Table S7. List of regulomes of inhibitory neurons in brains across 11 species. The TFs and corresponding target genes expressed by inhibitory neurons were shown in species including human, monkey, pig, hamster, hedgehog, civet, mink, chinchilla, alpaca, lizard and turtle. The TFs and target genes conserved in inhibitory neurons of at least 4 species are also presented here

Table S8. GO terms of target genes of TFs expressed by excitatory and inhibitory neurons in brains across 11 species including human, monkey, pig, hamster, hedgehog, civet, mink, chinchilla, alpaca, lizard and turtle
