# Supplementary figures and images for: Screening of cell‐virus, cell‐cell, gene‐gene crosstalk among animal kingdom at single cell resolution
Source: Clin Transl Med. 2022 Aug 2;12(8):e886. doi: 10.1002/ctm2.886 (PMC9345398; doi:10.1002/ctm2.886)

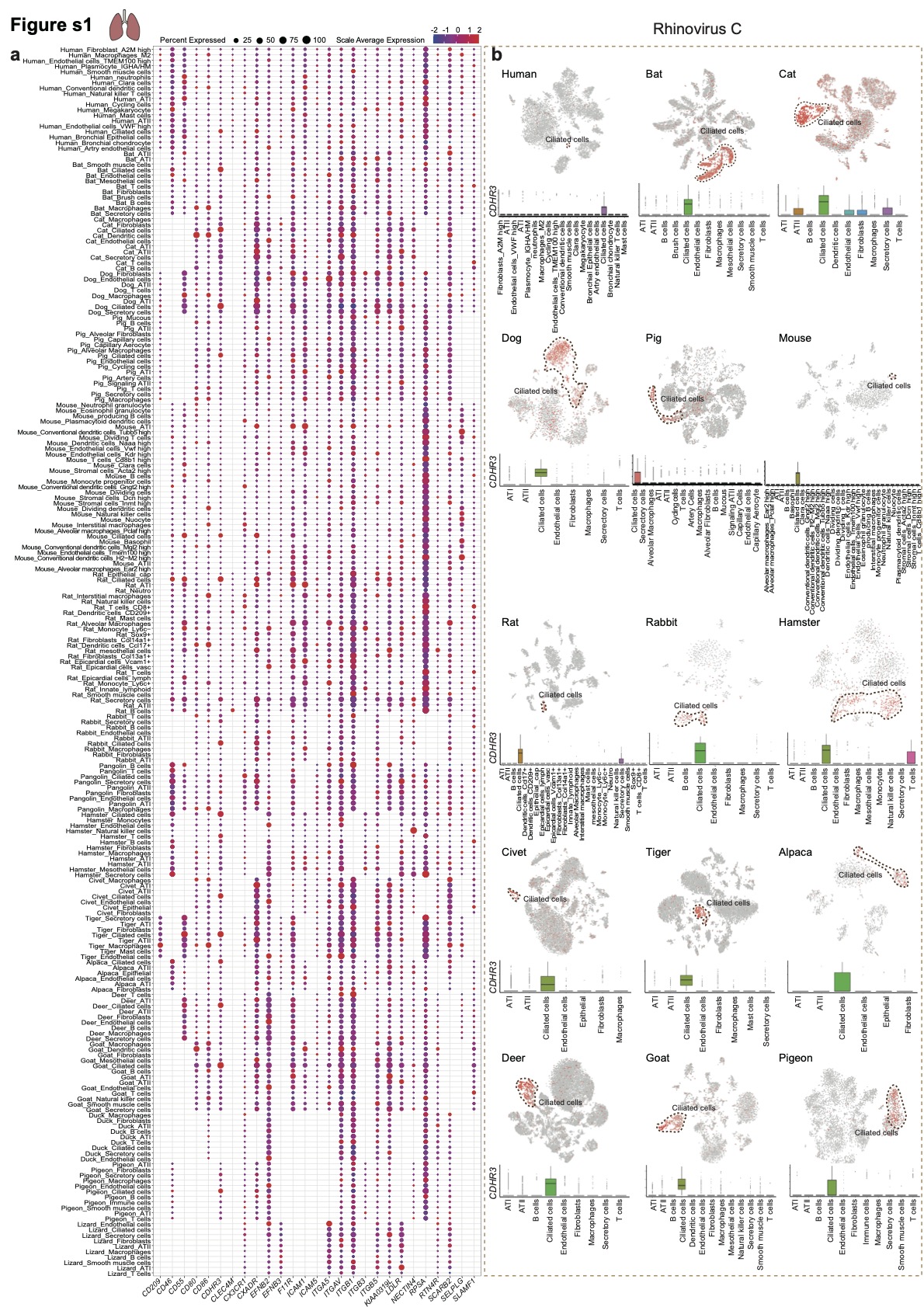

Supplement: Supplementary file 1 — Figure S1. Cross‐species screening of respiratory virus target cells in lung tissues. (A) The species‐split dot plot shows the screening of the expression of respiratory virus receptors in all cell populations profiled in lung tissues. Colour saturation of dots reflects the scaled average expression, while dot size indicates the percentage of cells of each cell type expressing the receptor. (B) The specific expression of CDHR3, a receptor for rhinovirus C of Picornaviridae, in ciliated cells across species [file CTM2-12-e886-s005.jpeg]
